# What is case management in palliative care? An expert panel study

**DOI:** 10.1186/1472-6963-12-163

**Published:** 2012-06-18

**Authors:** Annicka G M van der Plas, Bregje D Onwuteaka-Philipsen, Marlies van de Watering, Wim J J Jansen, Kris C Vissers, Luc Deliens

**Affiliations:** 1 Department of Public and Occupational Health, EMGO Institute for Health and Care Research, VU University Medical Center, Amsterdam, the Netherlands; 2 Palliative Care Center of Expertise, VU University Medical Center, Amsterdam, the Netherlands; 3 Kennemer Gasthuis, Haarlem, the Netherlands; 4 Department of Anaesthesiology, VU University Medical Center, Amsterdam, the Netherlands; 5 Agora, National Support Center for Palliative Terminal Care, Bunnik, the Netherlands; 6 Department of Anaesthesiology, Pain, and Palliative Medicine, Radboud University Nijmegen Medical Center, Nijmegen, the Netherlands; 7 End-of-Life Care Research Group, Ghent University and Vrije Universiteit Brussel, Brussels, Belgium

**Keywords:** Case management, End of life care, Palliative care

## Abstract

**Background:**

Case management is a heterogeneous concept of care that consists of assessment, planning, implementing, coordinating, monitoring, and evaluating the options and services required to meet the client's health and service needs. This paper describes the result of an expert panel procedure to gain insight into the aims and characteristics of case management in palliative care in the Netherlands.

**Methods:**

A modified version of the RAND®/University of California at Los Angeles (UCLA) appropriateness method was used to formulate and rate a list of aims and characteristics of case management in palliative care. A total of 76 health care professionals, researchers and policy makers were invited to join the expert panel, of which 61% participated in at least one round.

**Results:**

Nine out of ten aims of case management were met with agreement. The most important areas of disagreement with regard to characteristics of case management were hands-on nursing care by the case manager, target group of case management, performance of other tasks besides case management and accessibility of the case manager.

**Conclusions:**

Although aims are agreed upon, case management in palliative care shows a high level of variability in implementation choices. Case management should aim at maintaining continuity of care to ensure that patients and those close to them experience care as personalised, coherent and consistent.

## Background

Patients facing a life-threatening illness are likely to experience palliative care needs [1,2]. According to the World Health Organization (WHO), palliative care aims at improving the quality of life of patients and their families, through the prevention and relief of suffering by means of early identification and impeccable assessment and treatment of pain and other problems, physical, emotional, and spiritual [3]. Palliative care is complex care. Firstly because it demands attention to and knowledge of not only disease, pain and symptom management, but also a range of other non-medical issues from reimbursement structures to availability of social services and spiritual care [3]. Gaps in the general and specialist knowledge required by the health care provider must be filled by access to reliable knowledge from others. Secondly, communication plays a pivotal role; several professionals and informal caregivers across settings can be involved and round-the-clock continuity of information is necessary to deliver consistent care sensitive to rapidly changing needs. In 98% of their palliative care patients, Dutch General Practitioners (GPs) cooperate with at least one other caregiver, with a mean number of four [4]. In the Netherlands, about half of patients experience one or more transfers in their last month of life [5], implying the need for communication across settings at least at the start of the transfer period. This will probably be even more true in future with increasing life expectancy and a growing number of patients with multiple chronic diseases [6] resulting in, among other things, more health care needs and more need for the coordination of care.

Case management has developed as a means of ensuring continuity of care for patients with complex care needs. It is a heterogeneous term for care that consists of assessment, planning, implementation, coordination, monitoring and evaluation of the options and services required to meet the client's health and service needs [7]. It has been used for many years in psychiatry [8], among frail elderly people [9] and many other populations. There have been varying research results on its effectiveness. There are numerous models of and variations in ways of delivering case management [10]. Adding to the confusion is the multitude of names given to case management; care management, care coordination, disease management, and managed care being some of the most common in the nursing field. Most studies compare one application of case management with care as usual, there is little research comparing different models or applications of case management. It is difficult to compare studies due to differing methodologies and outcome measures, and unclear definitions and descriptions of case management [11-13]. Therefore, we conclude that based on current research, for most medical conditions there is no way of identifying the best model for delivering case management.

The same can be said for case management in palliative care. No reviews on case management in palliative care were found and there is no definitive evidence of its effectiveness in palliative care. Some positive results are reported. In a randomised trial among patients with advanced chronic obstructive pulmonary disease (COPD), congestive heart failure (CHF) or cancer, case management resulted in increased patient satisfaction with care and the earlier development of advance directives [14]. In patients with advanced illness (mostly cancer) receiving case management, compared with a matched historical control group, hospice use and number of hospice days increased [15]. There appear to be variations in the application of case management in palliative care. Differences can be seen in target populations (e.g. cancer only [16] or a range of diagnoses [17]), whether principles of disease management should be integrated [18] or focus should be solely on terminal care [19], whether case management should be delivered by a multidisciplinary team [20] or not [15] and a broad range of other variations. Again, these studies cannot be compared, therefore, no conclusions can be drawn as to which application of case management should be preferred.

The question of how case management should best be delivered in palliative care is unanswered. The purpose of this study was to formulate the aims of case management and describe essential characteristics of case management in palliative care in the Netherlands, as perceived by experts. The expert panel procedure also gave insight into which topics there is consensus between experts and what are the main differences in opinion between them.

## Methods

### Design

The RAND® / University of California at Los Angeles (UCLA) appropriateness method is developed to combine scientific evidence with the collective judgment of experts to yield a statement regarding the appropriateness of performing a procedure at the level of patient-specific symptoms, medical history and test results [21]. The aim of this method is to reach consensus on which medical procedures are appropriate in certain medical conditions and circumstances. With a modified version of this method it is possible to investigate whether there is consensus or disagreement for a diverse range of topics. In three written rounds we consulted experts to formulate and rate aims and characteristics of case management in palliative care. Purpose of round 1 and 2 was to formulate a list of aims and characteristics, in the third round experts rated the aims and characteristics on importance for successful implementation of case management in palliative care.

### Expert panel

We invited 73 experts with experience in palliative care to participate in the expert panel: general practitioners, coordinators of palliative care networks, case managers working in palliative care, researchers and policy makers in palliative care. The perspective of district nurses was included in the expert panel through case managers and scientists in the field of nursing. Two experts declined but proposed four others to take their places and the colleague of another was added leading to the questionnaire being sent to 76 experts. Of those, 46 (61%) participated in at least one round. Twenty-four experts gave their reasons for not participating: lack of time (n = 13), lack of knowledge about case management (n = 7), prolonged illness (n = 4). Four reactions in the first round and two in the second were not traceable because they were returned anonymously. This study is exempt from approval from an ethics committee.

### Selection of aims and characteristics

We drafted a first list of aims and characteristics of case management in palliative care based on information from existing initiatives, literature and previous research. We used four headings to partition our list of aims and characteristics: aims of case management in palliative care, characteristics of content of case management in palliative care, characteristics of structure of case management in palliative care and general conditions. The 16 characteristics in the fourth section, general conditions, related so commonly to care in general (e.g. 'the caseload is in ratio with the terms of employment of the case manager and the necessary time investment for individual patients') that they were omitted for the purposes of this paper.

For the aims of case management in palliative care, we made use of the conceptual framework of continuity of care by Bachrach [22]. She identified seven dimensions in continuity of complex care. The dimensions put together describe an ideal model for care in situations where several health care providers, settings and/or needs are involved. Case management does not necessarily incorporate all elements in itself, but its task is to make sure the patient receives continuity of care. Bachrach listed these dimensions specifically for people with long-term mental disorders, and we hypothesised that they would be useful as a starting point in identifying the aims of case management in palliative care. We reformulated the characteristics to reflect palliative terminology and discourse. Additional to the seven characteristics derived from Bachrach, we added two more, one specifically on palliative care (care or coordination of care is aimed at quality of life and death) and the other because the literature suggests that continuity of care across settings is problematic in palliative care [23,24] and we hypothesised that case management should pay special attention to that aspect. In Table 1 the dimensions of Bachrach and the aims of case management are reported.

**Table 1:** Transformation of dimensions of continuity of care to aims of case management in palliative care sent to the expert panel for feedback in round 1

	**Aims of case management, sent to the expert panel at start of round 1**	**Dimensions of continuity of care**
1	Delivery and/or coordination of care is aimed at quality of life and death (not at curing the patient)	
2	Care is longitudinal; it lasts for a minimum of two weeks and lasts as long as necessary	Continuity of care has a temporal dimension, it is longitudinal in nature; the patient’s treatment parallels his or her progress even though the individual health care provider, specific treatment modalities, or specific site of care may change. Episodes are consecutive and related
3	Care is individual: it is tailored to the individual needs of the patient	Continuity of care has an individual dimension, the care is planned with and for the patient and family with consideration for their specific needs
4	Care is flexible; it is adjusted to the pace of the patient. This means for example that the frequency of contacts can vary over time	Continuity of care is characterized by flexibility. A flexible service system relieves the patient of pressures that may be placed on him or her to exhibit ‘progress’ or to move ‘forward’ along a continuum. The flow in services should correspond to changes in the patient’s circumstances and needs
5	The relationship with the patient is central in care; the patient experiences care as familiar and close	Continuity of care has a relationship dimension, either in contacts with an individual provider or in an ‘institutional alliance’ in which the patient develops closeness with more than one service provider at a time. The patient is able to rely, over time, on having associations with a person or persons who are interested in him or her and who respond to him or her on a personal level
6	Care is comprehensive; the patient can receive a diverse array of care and support according to needs and wishes	Continuity of care as a cross-sectional dimension; it is comprehensive in a sense that it consists of a variety of services related to the many needs of the patient. It has a distinctly interdisciplinary quality.
7	Care is characterised by communication; between the case manager and the patient and between the case manager and other care providers communication is clear and sufficient	Continuity of care has a dimension of communication, both between the patient and service providers and among the various service providers involved in the care. One aspect of this is continuity in information
8	Care is accessible; the case manager can be reached and care is low-threshold and financially accessible	Continuity of care is characterized by accessibility, the patient will be able to reach the service system when she/he needs it and in a way in which she/he can handle, both psychologically and financially. The patient does not experience barriers to service delivery, whether they be of a physical, psychological, or economic nature. Implicit in this dimension is the patient’s access to 24-hour crisis intervention
9	Care is delivered at home or where the patient is staying	

### Procedure

In three written rounds the experts were asked to formulate and rate aims and characteristics of case management in palliative care. In the first round we presented the first draft of the list of aims and characteristics and the expert was asked to add and remove some, give textual feedback and feedback on the aims and characteristics included. For readability characteristics were clustered around themes within the sections; aims of case management in palliative care, characteristics of content of case management, characteristics of structure of case management, and general conditions. In the second round we sent a new draft based on the respondents' feedback, with the same question. No reaction was required if the participant agreed with the content and formulation. In order to be rated independently in the third round, the clusters were then divided into separate characteristics (see Table 2 for an example). Thus, a list of 41 clustered aims and characteristics was divided into 104 separate aims and characteristics. In the third round the expert panel rated all aims and characteristics on a nine-point scale, a score of one indicating that the aim or characteristic was 'not important for successful implementation' and of nine that it was 'very important for successful implementation' of case management in palliative care.

**Table 2:** Example of a clustered characteristic in round 2 and division into separate characteristics for round 3

**Clustered characteristic in round 2**	**Divided into separate characteristics in round 3**
2.5. Within a week of referral to case management, the case manager contacts the general practitioner and district nurse and other relevant professionals…	
yes, to reach an understanding on cooperation	2.5.a Within a week of referral to case management, the case manager contacts the general practitioner and district nurse and other relevant professionals to reach an understanding on cooperation.
yes, to match provision of care	2.5.b Within a week of referral to case management, the case manager contacts the general practitioner and district nurse and other relevant professionals to match provision of care.
yes, to gain relevant information	2.5.c Within a week of referral to case management, the case manager contacts the general practitioner and district nurse and other relevant professionals to gain relevant information.
other: ......................	2.5.d Within a week of referral to case management, the case manager contacts the general practitioner and district nurse and other relevant professionals for other than aforementioned reasons.
no	

### Data analysis

We calculated the mean, standard deviation, median and median absolute deviation (M.A.D.) for all aims and characteristics. Agreement was calculated according to the procedure described by the RAND Corporation specifically designed for expert panels with more than nine participants [21]. Thus, according to the RAND criteria, for an aim or characteristic to be considered important for successful implementation of case management two requirements for agreement had to be met:

1) the expert panel agreed with the aim or characteristic, meaning that an aim or characteristic was scored 7 to 9 by 80% of participants,

2) the expert panel agreed with each other, meaning that the Interpercentile Range Adjusted for Symmetry (IPRAS) is larger than the Interpercentile Range (IPR). We used .30 and .70 percentile scores to calculate the lower and upper limit of the IPR.

All other results are categorised as 'disagreement'. We used the M.A.D. as an estimator of dispersion to assess the level of disagreement within the expert panel. This measure is less susceptible to outliers than the standard deviation. To distinguish between a high and a moderate level of disagreement we used a cut off score of M.A.D. = 2.0.

## Results

### Round 1 and 2

In the first round we received 35 reactions on the aims and characteristics. In Table 3 the response is shown differentiated by the discipline of the participants. Main topics addressed by the experts on the first draft were: inclusion of informal caregivers (family, partner) in case management, communication and role delineation between the case manager and other health care professionals and the necessity of tailoring care to individual needs and wishes. Also, wording of the aims and characteristics was altered accordingly to feedback from the expert panel. This resulted in an adapted draft sent around for round two. In the second round we received 12 reactions on the adapted draft. The feedback on this draft mainly concerned suggestions for improvements in detail. The complete list of aims and characteristics for case management in palliative care formulated after round two is reported in Additional file 1.

**Table 3:** Background characteristics of respondents per round

	**Round 1**	**Round 2**	**Round 3**	**one or more responses**
Palliative care				
- case management	8	3	6	9
- coordinator of palliative care network	6	3	10	10
General Practitioners and other physicians	5	0	8	9
Other				
- research	9	4	9	11
- policy makers	3	0	1	3
Anonymous reply^1^	4	2	0	4
Total^2^	35	12	34	46

^1^ Some responses could not be traced, we are not certain whether the two unknown respondents from round two did or did not respond in round one. The total number may be between 4 and 6.

^2^ Some responses could not be traced, we are not certain whether the two unknown respondents from round two are unique, so the number of persons with one or more responses is between 46 and 48.

### Round 3

In the third round we received 34 reactions from the expert panel. Table 4 shows that agreement was reached on 35 aims and characteristics. Overall, about a third of the aims and characteristics met with agreement (34%), almost half with a moderate level of disagreement (49%), and less than a fifth (17%) with a high level of disagreement. Both aims and characteristics which are met with agreement and with a high level of disagreement are marked in the Additional file 1. There were no notable differences between experts from different backgrounds on rating the aims and characteristics (see the Additional file 1 for mean and median scores).

**Table 4:** Scoring of the aims and characteristics by the expert panel

**Section**	**Number of clustered characteristics**	**Number of separate characteristics**	**Characteristics of agreement (%)**	**Characteristics of moderate disagreement**	**Characteristics of high disagreement**
				**M.A.D. < 2**	**M.A.D. ≥ 2**
Aims	10	10	9 (90%)	1 (10%)	0
Content	20	48	19 (40%)	21 (44%)	8 (17%)
Structure	11	46	7 (15%)	29 (63%)	10 (22%)
Total	41	104	35 (34%)	51 (49%)	18 (17%)

### Aims of case management in palliative care

In section one on aims almost all aims were met with agreement (90%) and none with a high level of disagreement. The one aim with a moderate level of disagreement (Additional file 1, aim 1.2) used the term ‘care on demand’ ('vraaggestuurd'), which is used by Dutch policy makers to indicate that the patient is central to care as opposed to 'care as supplied' ('aanbod gestuurd') which prioritises the habits, rules and regulations of the institution delivering it. This characteristic was added at the request of some of the experts because they felt that aim 1.4 on individual care did not adequately cover the aspect of care on demand. However, we received questions on this term (e.g. 'does this mean that care should not be proactive?') that made clear that the denotation of the term is not well known among the expert panel. At the same time we received feedback indicating that the expert panel agrees that the patient should be at the centre of care and that it should be tailored to the individual needs of the patient and aim 1.4 was met with a high level of agreement.

### Content of case management in palliative care

In section two on content of case management most characteristics were met with a moderate level of disagreement (44%), while another 40% were met with agreement and a small proportion with a high level of disagreement (17%). Within this section the highest level of disagreement (M.A.D. = 2.33) was on nursing care tasks (characteristic 2.1.a). This stems from the opinion of some experts that the number of health care providers surrounding the patient should be kept as low as possible. The district nurse can perform case management next to other duties. Others believe that district nurses, due to their busy schedules, do not have time to offer patients adequate comfort, reassurance and information and this will take second place to their nursing tasks. Comfort, reassurance and information may also be needed by patients who are not yet using care from a district nurse.

### Structure of case management in palliative care

In section three on structure of case management most characteristics were met with a moderate level of disagreement (63%), while 22% encountered a high level of disagreement and only 15% were met with agreement. Within this section there were three characteristics with a joint highest level of disagreement (M.A.D. = 2.24): whether the case manager should combine case management with other tasks (e.g. consultation) (characteristic 3.5.b), whether she or he should be accessible 24 hours a day, seven days a week (characteristic 3.8.a), and if the target group she or he works for includes all patients with a life-threatening disease (characteristic 3.7.c).

## Discussion

This study shows that agreement was high on the aims of case management. However, how case management should be implemented, and exactly which elements of care it should include, is more open for debate. Disagreement was highest on topics regarding whether the case manager should perform hands-on nursing care themselves or not, on the target group, on accessibility of the case manager and on performance of other tasks besides case management.

### Strengths and limitations

This is the first study using a structured procedure to report on the importance of the aims and characteristics of case management in palliative care. The expert panel reflects the opinions of case managers, coordinators of palliative care networks, general practitioners and other physicians, researchers and policy makers. There were no marked differences between experts from different backgrounds on rating the aims and characteristics. However, these opinions not necessarily reflect practice and we lack information on how often and how case management is implemented in the Netherlands. Also, our results may only be representative for mixed public-private health care systems with a strong primary care gatekeeper that resemble the Dutch system. The characteristics of case management may be different in other health care systems.

### Aims of case management in palliative care

The aims that met agreement are in accordance with the general principles of palliative care and also reflect the patient advocacy model of case management [25]. This model offers comprehensive coordination of services aimed at quality of care and is distinguished from the interrogative model, which is more focused on clinical decision-making and emphasises cost-effectiveness. The aims also underline the importance of the seven dimensions of continuity of care formulated by Bachrach for psychiatric care [22]. This conceptual framework appears to be valid for complex continuous care in general, whether it is psychiatric care or palliative care.

### Content of case management in palliative care

Translation from aims to content of care is apparently relatively straightforward, with 40% agreement and only 17% strong disagreement on what care should be included. Offering information and support, identifying needs and adjusting care to match the patient's needs are the main tasks of the case manager. This can also be seen in descriptions of case management in palliative care [20,26], for cancer patients [27] and in a Delphi study on case management for patients with dementia [28]. Delivery of hands-on patient care is the most important area of disagreement within the expert panel. As mentioned in the results section, this stems from task alignment between the district nurse and case manager and whether these should be two different people or not. Besides, this also touches on the discussion whether palliative care should be part of primary (generalist) care, delivered by specialised palliative care providers, or in a cooperation between the two [29]. Case management could be delivered in a multidisciplinary team taking over all care, or case management can be guiding and assisting the primary health care providers (GP and district nurse) in their care for the patient. Another notable topic of disagreement is whether case management should stop before bereavement support is provided. The panel agrees that bereavement support is part of palliative care, reflected in agreement with characteristic 2.18.c. and aim 3. Whether there can be other endpoints for case management may be related to the target group, which is also a point of disagreement for the expert panel (reflected by characteristics 3.7 a, b and c). In a mixed-method study on case management for cancer patients, there are two distinct case management trajectories for patients receiving curative care and those receiving palliative care [27]. For curative patients case management can be short-term and stops when information needs are met. The discussion on bereavement support may also be a reflection of the Dutch reimbursement system, where it is not financed by public means and therefore any time the case manager spends on delivering it is not compensated.

### Structure of case management in palliative care

Translation from aims to structure of case management is apparently less straightforward, with only 17% agreement and 22% strong disagreement. Characteristics such as the target group and the accessibility of the case manager may reflect the scope and depth of the case manager’s task: when can she or he work with the patient themselves and at what point does she or he refer to another professional? In the aforementioned Delphi study on case management for patients with dementia, no agreement could be reached on similar topics [28]. Apparently, in correspondence with applications of case management in cancer [11], CHF [12] and dementia [13,28], also in palliative care there is no unique best way to deliver case management according to experts.

## Conclusions

Case management in palliative care should aim at maintaining continuity of care to ensure that patients and those close to them experience palliative care as personalised, coherent and consistent. There is a high level of agreement about the underlying dimensions of continuity of care [22]. The most important issues in implementation preferences are defining the target group of case management, the performance of other tasks besides case management, accessibility of the case manager and delivery of hands-on nursing care by the case manager. Research into the feasibility of different options and their effects on implementation could help health care planners make informed decisions on the best way to deliver case management.

## Competing interests

The authors declare that they have no competing of interest.

## Authors’ contributions

AvdP participated in the design of the study, carried out the measurements, analysed and interpreted the data, and drafted the manuscript. BO-P conceived of the study, participated in its design and coordination and made substantial contributions to the data interpretation and writing of the paper. MvdW, WJ, KV and LD participated in design of the study, interpretation of the data and critical revision of the manuscript for important intellectual content. All authors read and approved the final manuscript.

## Pre-publication history

The pre-publication history for this paper can be accessed here:

http://www.biomedcentral.com/1472-6963/12/163/prepub

## Supplementary Material

Additional file 1 Aims and characteristics of case management in palliative care. This file contains a full list of all aims and characteristics of case management in palliative care, as formulated and rated by the expert panel. (PDF 129 kb) Click here for file
