# Supplementary material for: What is case management in palliative care? An expert panel study
Source: BMC Health Serv Res. 2012 Jun 18;12:163. doi: 10.1186/1472-6963-12-163 (PMC3413598; doi:10.1186/1472-6963-12-163)
Supplement: Additional file 1 — Aims and characteristics of case management in palliative care. This file contains a full list of all aims and characteristics of case management in palliative care, as formulated and rated by the expert panel. (PDF 129 kb) [file 1472-6963-12-163-S1.pdf]

Additional file: Appendix

Appendix - Aims and characteristics of case management in palliative care

In the three tables below all aims and characteristics that resulted from round 1 and 2 of the expert panel are listed. In round 1 and 2 all aims and characteristics were clustered, for round 3 they were divided into separate aims and characteristics. The ratings from round 3 are in de columns behind the aims and characteristics. Some of the separated aims and characteristics were not rated by the expert panel, those cells remain empty (e.g. in the case of contact with those next to the patient after the patient is deceased, the option 'yes, to evaluate and to offer bereavement support' is not put before the expert panel because they already rated the evaluation and bereavement support separately).

Table 1. Aims of case management in palliative care[1]

	**Ratings Palliative Care (n=16)**		**Ratings GPs and other physicians (n=8)**		**Ratings Other (n=10)**		**Ratings Total** (n=34)
	mean	median	mean	median	mean	median	M.A.D.
1.1. Care is aimed at quality of life and death*	8.4	9	8.8	9	8.3	9	0.53
1.2. Care is offered on demand, care will only be delivered in agreement with and according to the wishes of the patient and informal support system	6.9	8	5.4	6	6.1	7	1.76
1.3. Care is longitudinal; it starts when needed and lasts until	7.8	8	8.0	8	8.2	8.5	0.88

the patient is deceased and the informal support system has received bereavement support*							
1.4. Care is tailored to the individual needs and wishes of the patient and informal support system*	8.4	9	8.4	8	8.8	9	0.50
1.5. Care is flexible; content, duration and frequency of contacts are adjusted according to the needs of the patient and informal support system*	8.5	9	8.0	8	8.6	9	0.58
1.6. The relationship with the patient and informal support system is familiar, close and personal*	8.5	9	7.8	8	8.5	9	0.70
1.7. Care is comprehensive; the patient and informal support system receive a diverse array of care and support according to their needs and wishes*	7.9	8	7.5	7.5	8.0	8	0.72
1.8. Communication is a cornerstone; there is ongoing sufficient and clear communication between the case manager, the patient and the informal support system and between the different care providers working with and for the patient*	8.8	9	7.9	8	8.4	9	0.53

1.9. Care is accessible and low-threshold; clear arrangements are made for round-the-clock care and it is financed through health care insurance (or other arrangement without costs to the patient), no referral is needed*	8.2	9	8.4	8	8.4	9	0.71
1.10. Care is primarily delivered in the community and follows the patient throughout the process of illness regardless of place of stay of the patient*	7.9	8	7.6	8	7.5	7.5	0.94

[1] For indication of agreement and high level of disagreement, the following symbols are used:

* agreement is reached on importance of this element for successful implementation of case management in palliative care

^ high level of disagreement is reached on importance of this element for successful implementation of case management in palliative care

Table 2. Characteristics of content of case management in palliative care[2]

	**Ratings Palliative Care (n=16)**		**Ratings GPs and other physicians (n=8)**		**Ratings Other (n=10)**		**Ratings Total** (n=34)
	mean	median	mean	median	mean	median	M.A.D.
2.1. The case manager:							
(a) provides hands-on patient care, including technical nursing interventions^	3.2	3	5.0	5	5.2	5	2.33
(b) does not provide hands-on patient care^	6.7	7	3.9	3.5	3.7	4.5	2.12
2.2. At the start the case manager draws up a care plan together with the patient and his/her informal support system. This care plan is part of the (medical) record of the patient. (yes / no)	6.2	7	6.1	6	7.1	7	1.26
2.3. The care plan describes the following:							
(a) the mental and physical health of the patient^	5.4	6	4.5	4.5	7.1	7.5	2.26

(b) the social and spiritual wellbeing and needs and preferences of the patient and informal support system	6.1	6.5	5.0	5	7.7	8.5	1.94
(c) the abilities, skills and limitations of the informal support system	6.1	7	5.4	7	7.5	7.5	1.82
(d) an assessment of possibilities and preferences with regard to care of patient and his/her informal support system (e.g. preferred place of care, preferred place of death)	6.4	7	6.2	7	7.8	8.5	1.59
(e) the aims and content of care to be received	6.3	7	5.7	6	7.9	7.5	1.59
(f) an agreed list of people responsible for all aspects of care and information on how and where to reach them	7.3	8	6.7	7	7.9	8.5	1.38
(g) the name and contact information of an authorised agent who acts and decides on behalf of the patient when necessary	7.0	8	7.0	7	8.1	9	1.35
2.4. The care plan is regularly evaluated by the case manager, the patient and his/her informal support system and adjusted if necessary (according to an established procedure outlining how and when this is done). The amendment is part of the	6.8	8	6.0	6	8.1	9	1.44

patient file. (yes / no)							
2.5. Within a week after referral to case management, the case manager gets in contact with the general practitioner and district nurse and other relevant professionals							
(a) yes, to reach an understanding on cooperation*	7.8	8	7.1	8	7.3	7.5	1.06
(b) yes, to match provision of care*	8.3	9	6.3	7	7.8	8	1.09
(c) yes, to gain relevant information*	7.2	8	6.6	7.5	7.4	7	1.36
(d) no							
(e) other:							
2.6. The case manager acts as an advocate for the patient and his/her informal support system. If the patient and his/her support system do not agree with each other on issues, the case manager strives to reconcile the needs and preferences. (yes / no)	7.6	8	6.1	6.5	7.9	8.5	1.09
2.7. The case manager clarifies treatment and care options and discusses who is responsible for treatment and care. * (yes /	7.4	8	6.8	7.5	8.1	8.5	1.15

no)							
2.8. The case manager assists the patient and his/her informal support system in making decisions on treatment, care and support by giving information and by discussing significant thoughts and emotions. * (yes / no)	8.0	8	6.6	7.5	8.4	9	1.12
2.9. The case manager has solid knowledge of relevant services and organisations and offers complete and independent information tailored to the needs and preferences of the patient and his/her support system. * (yes / no)	8.5	8.5	6.8	7	8.6	9	0.82
2.10. The case manager matches the patient and his/her support system to necessary and preferred care and services or aids the patient and support system in organising care.* (yes / no)	8.3	8	6.6	7	8.5	9	0.85
2.11. The case manager follows the course of illness and the situation of the patient and his/her informal support system, anticipates problems and tries to prevent crises.* (yes / no)	7.9	9	6.6	7.5	8.6	9	1.18

2.12. The case manager monitors progress and coordination of care and secures and writes down agreed courses of care. (yes / no)	7.6	8	6.1	7	8.5	8.5	1.21
2.13. The case manager clarifies financial matters:							
(a) yes, with regard to applications for care, if and when their own contribution is required, and on procedures for compensation or allowance.	6.8	7	6.1	7	7.8	8	1.29
(b) yes, with regard to income, pension, inheritance and funeral costs.^	4.9	6	4.3	4.5	5.5	5	2.12
(c) no, but the case manager aids in referral to a specialist if desired	7.0	7	5.4	6	5.8	6	1.38
(d) no							
2.14. With regard to nursing treatment the case manager should:							
(a) identify the needs and preferences of the patient and his/her informal support system*	7.6	8	6.6	7	8.2	8.5	1.06

(b) give information and support to the patient and his/her informal support system	7.3	7	6.4	7	8.2	8	1.12
(c) organise care	7.5	8	6.3	6.5	8.1	8	1.21
(d) give care^	3.4	4	3.0	2	5.5	5.5	2.09
2.15. With regard to social and personal wellbeing of the patient and his/her informal support system the case manager should:							
(a) identify the needs and preferences of the patient and his/her informal support system*	8.1	8	6.6	7.5	8.5	8.5	0.85
(b) give information and support to the patient and his/her informal support system*	7.7	8	6.4	7	8.3	8.5	1.03
(c) organise care*	8.1	8	5.9	6.5	8.3	8	1.09
(d) give care^	5.9	6	3.3	2.5	5.2	5.5	2.06
2.16. With regard to spiritual care the case manager should:							
(a) identify the needs and preferences of the patient and his/her informal support system*	8.2	8	6.6	7.5	8.4	8	0.76

(b) give information and support to the patient and his/her informal support system*	7.7	8	6.1	7	8.0	8	1.06
(c) organise care*	8.0	8	6.0	7	8.1	8	1.09
(d) give care	5.4	6	3.0	2.5	4.5	5	1.91
2.17. With regard to practical help / assistance the case manager should:							
(a) identify the needs and preferences of the patient and his/her informal support system*	8.1	8	7.0	8	8.3	8.5	0.88
(b) give information and support to the patient and his/her informal support system*	7.8	8	6.3	7	8.4	8.5	0.94
(c) organise care*	7.6	8	6.0	6.5	8.0	8	1.24
(d) give care	3.9	3.5	3.0	2.5	4.2	5	2.00
2.18. Care from the case manager ends when:							
(a) a well-functioning network of care providers is put in place for the patient and his/her informal support system^	5.7	6	2.5	2	3.5	3	2.24
(b) the patient is deceased^	4.0	3	4.4	4.5	3.6	3.5	2.03

(c) the patient is deceased and bereavement support for his/her informal support system is in place or has been delivered*	7.8	8	6.9	7.5	8.0	8.5	1.26
2.19. One or more contacts with the patient's informal support system takes place between one and eight weeks after the patient is deceased.							
(a) yes, to evaluate care and case management	7.3	8	6.1	7	7.3	7	1.18
(b) yes, to offer bereavement support	6.5	7	5.8	7.5	6.3	6.5	1.85
(c) yes, to evaluate and to offer bereavement support							
(d) no							
2.20. Within two weeks of the patient being deceased the case manager contacts the general practitioner and district nurse and other care professionals.							
(a) yes, to evaluate care and case management	6.8	7	6.8	8	6.6	7	1.24
(b) yes, to evaluate cooperation	6.6	7	6.8	8	6.8	7	1.29
(c) yes, to evaluate care and cooperation							
(d) no							

[2] For indication of agreement and high level of disagreement, the following symbols are used:

* agreement is reached on importance of this element for successful implementation of case management in palliative care

^ high level of disagreement is reached on importance of this element for successful implementation of case management in palliative care

Table 3. Characteristics of structure of case management in palliative care[3]

	**Ratings Palliative Care (n=16)**		**Ratings GPs and other physicians (n=8)**		**Ratings Other (n=10)**		**Ratings Total** (n=34)
	mean	median	mean	median	mean	median	M.A.D.
3.1. The case manager works for:							
(a) a home care organisation	5.5	6.5	6.1	6	5.6	5	1.67
(b) a hospital^	3.9	4.0	2.8	2	4.9	5	2.09
(c) a hospice	4.4	5	4.6	5	3.6	4	1.67
(d) a residential care home	3.0	3	3.0	3	3.3	4	1.53
(e) a general practitioner or health care centre	4.3	5	5.4	5.5	4.1	5	1.88
(f) a specialised consultation team in palliative care	7.1	7.5	6.0	6	4.1	5	1.73
(g) a multidisciplinary support team	6.7	7	6.1	6.5	4.7	5	1.88
(h) a cooperative/network of palliative care providers	7.7	8	4.6	5	6.0	6	1.90
3.2. Referral to case management can be done by:							

(a) a home care organisation*	8.1	8	6.5	8	8.2	8.5	1.00
(b) a hospital*	8.1	8	7.5	8	8.5	9	0.79
(c) a hospice*	7.3	8	7.5	8	7.5	8.5	1.35
(d) a residential care home	7.2	8	7.1	8	7.3	8.5	1.45
(e) a general practitioner or health care centre*	8.4	8.5	7.6	8.5	8.0	9	0.91
(f) family/friends/the patient*	8.3	8	5.4	8	7.5	8.5	1.38
3.3. The case manager:							
(a) is part of a team of case managers	7.0	7	6.1	7.5	7.4	8	1.62
(b) is part of a multidisciplinary team	7.3	8	7.4	8	6.6	7.5	1.41
(c) other:							
3.4. The following disciplines are part of the multidisciplinary team or are otherwise available to the case manager for consultation:							
(a) a medical specialist^	6.6	7	4.4	3.5	6.3	7	2.03
(b) a general practitioner	7.6	8	7.5	8.5	7.3	8	1.36
(c) a psychologist	6.3	7	6.1	7	6.7	7.5	1.71

(d) a psychiatrist	4.4	4	3.0	2.5	4.7	5	1.61
(e) a spiritual counsellor	6.6	7	5.9	7	6.3	7	1.62
(f) a pharmacist^	4.3	4	5.0	5.5	5.3	5	2.03
(g) a physiotherapist	4.4	4.5	4.9	5.5	4.3	5	1.85
(h) a dietician	4.1	4	4.6	5	4.7	5	1.85
(i) a social worker	5.5	6	5.1	5.5	6.2	7	1.85
(j) an ethnicity consultant	4.9	5	4.9	5	4.0	5	1.82
(k) Centre for Youth and Family (knowledge centre for answers and advice on issues concerning upbringing, parenting, and growing up)	3.9	4	3.1	3	4.4	5	1.63
(l) when a case manager is part of a team of case managers only, arrangements for collaboration with other disciplines are made*	7.2	8	7.1	8	8.4	9	1.18
3.5. Case management is delivered:							
(a) as part of a larger/diverse range of tasks (e.g. consultation of nursing tasks, not combined with case management)	6.8	8	5.8	6	6.0	5.5	1.72

(b) by a case manager solely performing case management^	5.0	5.5	3.0	2.5	4.0	4.5	2.24
3.6. The case manager is one of the following:							
(a) nurse trained at level 4	4.1	3	4.6	4	3.1	4	1.81
(b) nurse trained at level 5	7.3	8	6.5	6.5	5.3	5	1.82
(c) nurse with further schooling in palliative care, oncology or another relevant field of specialist care	7.7	9	6.4	7	7.7	8.5	1.50
(d) nurse practitioner/clinical nurse specialist	5.9	6.5	6.3	7	7.4	7.5	1.68
(e) practice nurse in a general practice	4.3	4.5	5.5	6.5	4.7	5	1.97
(f) general practitioner^	4.8	5.5	6.5	8	4.4	5	2.12
(g) social worker	4.1	4	2.9	2	2.7	2	1.94
(h) other:							
3.7. Case management is for:							
(a) patients in need of palliative care (i.e. when there is no ongoing treatment aimed at curing disease or prolongation of life)^	5.8	5.5	3.6	2.5	3.2	3.5	2.09
(b) patients in need of palliative care or care aimed at	6.8	7.5	5.1	5	6.1	7	2.03

prolongation of life (i.e. when there is no ongoing treatment aimed at curing disease)^							
(c) patients with a life threatening decease (i.e. when care can be aimed at palliation, curing disease or prolongation of life or a combination of these aims)^	5.9	6	6.8	8	5.3	5	2.24
3.8. The case manager is accessible:							
(a) 24 hours a day, 7 days a week^	4.8	3.5	4.6	4	6.7	7	2.24
(b) office hours, 5 days a week, with a arrangement for times of crises	7.2	8	7.8	8	6.6	7.5	1.38
(c) office hours, 5 days a week^	5.9	6	4.4	4.5	3.7	4.5	2.03
(d) other:							
3.9. As a fundamental principle case management complements other care, it does not duplicate services or take over care of other providers. (yes / no)	8.5	9	6.6	8	6.0	6	1.34
3.10. The case manager engages in casefinding, for example by participating in team meetings in the local hospital. (yes /	7.4	7.5	5.4	5.5	7.2	7.5	1.62

no)							
3.11. The case manager identifies deficiencies in local care for patients in need of palliative care and discusses them with the regional coordinator of palliative care services.* (yes / no)	7.9	8	7.8	8	7.9	9	0.94

[3] For indication of agreement and high level of disagreement, the following symbols are used:

* agreement is reached on importance of this element for successful implementation of case management in palliative care

^ high level of disagreement is reached on importance of this element for successful implementation of case management in palliative care
